# Investigating role of abiotic side and finding optimum abiotic condition for improving gas biodesulfurization using *Thioalkalivibrio versutus*

**DOI:** 10.1038/s41598-022-10430-6

**Published:** 2022-04-15

**Authors:** Reza Peighami, Ehsan Motamedian, Behnam Rasekh, Fatemeh Yazdian

**Affiliations:** 1 Biotechnology Group, Department of Chemical Engineering, Tarbiat Modares University, Tehran, Iran; 2 Environment and Biotechnology Research Division, Research Institute of Petroleum Industry, Tehran, Iran; 3 Department of Life Science Engineering, Faculty of New Science and Technology, University of Tehran, Tehran, Iran

**Keywords:** Environmental chemistry, Environmental impact

## Abstract

Hydrogen sulfide (H_2_S) is a super toxic substance that produces SO_x_ gases when combusted. Therefore, it should be removed from gas streams. Biodesulfurization is one of the developing methods for removing sulfide. Gas biodesulfurization must be accelerated to be competitive with chemical processes. This process has two sides: biotic and abiotic sides. To increase the rate of sulfide removal, this substance should be given to the bacteria in the maximum amount (Max. − R_HS B_). Therefore, it is necessary to minimize the rate of adverse abiotic reactions of sulfide (Min. − R_HS A_). Minimizing the sulfide reaction with biosulfur and oxygen and thiosulfate generation (Min. − R_HS thio2_) was assessed in de-microbized medium. It was concluded that the pH should be kept as low as possible. The kinetics of thiosulfate formation from sulfide oxidation (− R_HS thio1_) are strongly dependent on the sulfide concentration, and to minimize this reaction rate, sulfide should be gently injected into the culture. To minimize sulfide reduction to hydrogen sulfide (Min. − R_HS rev_), the pH should be kept as high as possible. Using the Design Expert v.13, a model was driven for the abiotic side to obtain optimum condition. The pH value was found to be 8.2 and the sulfide concentration to 2.5E−05 M. *Thioalkalivibrio versutus* cultivation under identified abiotic conditions resulted in biological removal of sulfide up to 1.5 g/h. The culture was not able to remove 2 g/h input sulfide, and to increase this, the biotic side should be studied.

## Introduction

Hydrogen sulfide (H_2_S) is a toxic, corrosive, and foul-smelling gas. The products of its combustion are SO_x_ gases that are toxic and lead to acid rain. This gas (hydrogen sulfide) exists in natural gas streams, biogas, and gas produced in oil refining companies^1^. Typically, H_2_S is removed by chemical–physical methods, such as amine Claus, scavengers and liquid iron-based technologies, Lo-cat^2^. Also, recently new methods are developed to obtain valuable products from Hydrogen sulfide. Among these, the newly developed green and mild method to convert H_2_S into mercaptan alcohols by the addition reaction with epoxide mediated in tertiary amine-functionalized protic ionic liquids (PILs)^3^ and applying series of task-specific ionic liquids were developed and presented as both absorbents and catalysts for simultaneous capture and conversion of H_2_S into high valuable mercaptan acids using unsaturated acids as starting materials^4^ could be counted. Today, among various desulfurization methods that are available to remove hydrogen sulfide (H_2_S), environmentally friendly biological technology is the cheapest and simplest operation^5^. In such technologies, the sulfide-containing alkaline solution is sent to a biological reactor, where the sulfide is oxidized to elemental sulfur by sulfur oxidizing bacteria (SOB)^6^. The biodesulfurization process is extensively used in the oil and gas, food, paper and mining industries^7^. In this process, SOB converts soluble sulfide (HS^−^) and dissolved oxygen (O_2_) to elemental sulfur^8^. Several bacteria are reported to oxidize sulfide, among which *Thioalkalivibrio versutus* is reported to have the most capability^9^. In the process, the formation of undesired thiosulfate is a reason for decreasing the selectivity for the formation of valuable product sulfur^10^. Experimental studies show that abiotic oxidation of sulfide can produce thiosulfate^11–14^. Additionally, polysulfide ions abiotically could be oxidized to thiosulfate^15^. This means that in addition to biological reactions in the biodesulfurization process, there are several abiotic reactions that could directly and indirectly affect the biological side of the process^6,16,17^.

Therefore, there are two sides in the process: biotic and abiotic. Increasing the capacity of biodesulfurization for the process to be competitive with the chemical processes means maximizing the rate of biological sulfide conversion to sulfur, − R_HS B._ This would happen when sulfide inlet is gone to the biological side maximally. The balance of sulfide can be written as R_HS in_ + R_HS B_** + **R_HS A_ = d[HS^−^]/dt. where R_HS_ is the rate of inlet sulfide, R_HS B_ biologically converts sulfide and R_HS A_ represents abiotic sulfide conversion. R_HS A_ is the sum of three reaction rates: sulfide conversion to hydrogen sulfide (the reverse reaction of hydrogen sulfide solution), − R_HS rev_, sulfide reaction with oxygen to form thiosulfate, − R_HS thio1,_ sulfide reaction with elemental sulfur and oxygen to produce thiosulfate, − R_HS thio2_.

1 $$- \,{\text{R}}_{{\text{HS A}}} = - \,{\text{R}}_{{\text{HS rev}}} - {\text{R}}_{{\text{HS thio1}}} - {\text{R}}_{{\text{HS thio2}}}$$

To optimize the process, − R_HS B_ should be maximized. For this reason, d[HS^−^]/dt should tend to zero, and − R_HS A_ should be minimized. To minimize this term, the whole terms on the right side of Eq. (1) should be minimized. These reactions have been somewhat studied separately^13,18–20^, and all the reported works were performed in synthetic solution (e.g., by the addition of sodium polysulfides to distilled water or using elemental sulfur instead of biological sulfur). Hence, the lack of comprehensive work is felt in the real environment.

According to what was mentioned and considering the role of protons in both abiotic and biotic reactions of biodesulfurization, in the current work, the abiotic side of biodesulfurization was analyzed. In this way, the whole experiment proceeded in this research and could be divided into three parts: Part one analyzes the pH effect on hydrogen sulfide release and thiosulfate formation from sulfide at different pH values and oxygenation rates. Part two analyzes the effect of pH and oxygenation on thiosulfate formation from a mixture of sulfide and elemental sulfur. To proceed with the former part, the experiments were developed in a mixture of water and sulfide. The latter part proceeded in the de-microbized (decellularized) medium of *Thioalkalivibrio versutus* culture. The results show the optimum conditions for the abiotic side of the biodesulfurization process, which could affect the fate of the process and help to identify points that would be utilized to upgrade and maximize elemental sulfur selection. Part three involved culturing *Thioalkalivibrio versutus* and applying the conditions to control abiotic reactions. In this part, the results of two previous parts are processed using Design Expert software (v.13). According to this, a statistical model was developed and the optimum condition which was extracted using the model was applied to the culture medium to control the undesired formation of thiosulfate and release of sulfide as H_2_S. In this way, all the sulfide is given to the bacteria. Further efficiency and rate of sulfide utilization relate to the performance of bacteria (biotic side), in which protons again have a vital role. The biological reactions and role of protons in biochemical reactions were studied by the group and are reported elsewhere, which is beyond the scope of the current work.

## Material and methods

As pointed out in the previous section, the experiments of this work are done in three main parts. Part one lacks the presence of biosulfur (biologically produced elemental sulfur), part two is done in de-microbized medium and part three is in which abiotic conditions are applied to the *T. versutus* culture. All experiments were developed to clarify the significant role of protons and oxygen in the final state of the process.

### Part one: effect of pH on H_2_S release and oxygen on thiosulfate formation

As explained above, when sulfide is added to a solution, several reactions occur. All the reactions happen to equilibrium protons. pH is a sign of a proton state in a solution. Therefore, pH has a direct and significant effect on reactions and final equilibrium. To understand and determine the effect of pH on solution behavior, an experiment was designed and developed. For this reason, 5 g of sodium sulfide trihydrate (Na_2_S.3H_2_O) (Acros Organics, Belgium) was dissolved in 500 ml of bidistilled water as a stock. Because of hazards associated with H_2_S, Na_2_S was chosen as an analog for hydrogen sulfide. Six 100 ml solutions with different pH values of 2, 6, 7, 8, 9 and 12 were prepared based on bidistilled water. The pH was adjusted by adding HCl (5 N) and KOH (0.1 M) solutions to bidistilled water, and the pH was read by a Corning pH meter 240. As Na_2_S dissolved in water, the following reaction takes place:

2 $$Na_{2} S + H_{2} O \to 2 Na^{ + } + HS^{ - } + OH^{ - }$$

This reaction (Eq. 2) completely progresses to the right side, and it is expected to increase the pH in all six solutions. However, the main goal is to determine the behavior and fate of sulfide in the solution because in the natural gas biodesulfurization process, there is no such reaction, hydrogen sulfide (H_2_S) is directly added to the solution, and *HS*^−^ is formed. Therefore, the behavior of sulfide ions (HS^−^) is important, and the effect of pH will be investigated.

Regarding the solution and concentration of substances, HS^−^ could undergo different processes. One of the possible reactions that could occur and is not studied in detail is the conversion of sulfide ions to hydrogen sulfide (Eq. 3):

3 $$HS^{ - } \left( {aq} \right) + H^{ + } \left( {aq} \right) \leftrightarrow H_{2} S \left( {aq} \right)$$

4 $$H_{2} S \left( {aq} \right) \leftrightarrow H_{2} S \left( g \right)$$

**Figure 1:**
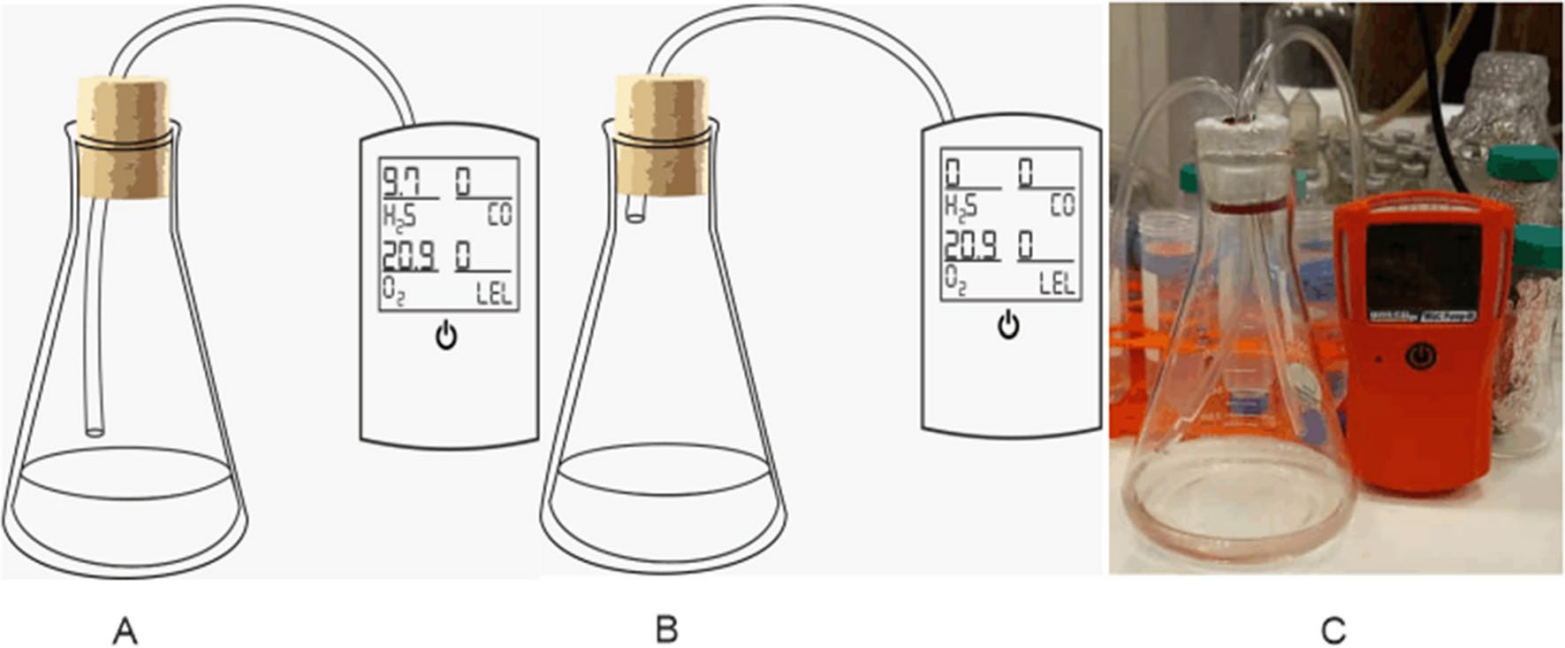
The assembled setup for H_2_S detection. (**A**) Schematics when the pipeline is in the bottom just near the liquid surface. (**B**) Schematic when the pipeline is at the top of flask. (**C**) The image of setup.

Conversion of sulfide ions to hydrogen sulfide consumes protons, and the occurrence of Eq. (3) strongly depends on proton capability of solution. To better understand the effect of pH on hydrogen sulfide formation, a set as seen in Fig. 1 was assembled. Six 100 ml solutions with different pH values were transferred to six 500 ml Erlenmeyer flasks connected to a H_2_S meter (Gas Clip, MGC Pump, USA). For each Erlenmeyer flask, 100 μl of stock Na_2_S solution (120 µM) was added, and the amount of released gaseous hydrogen sulfide in the empty space above the liquid in two places, just right the liquid surface and on the top of the flask at different times. Additionally, the final pH was read, and the remaining total sulfide (S_t_^2−^, which contains H_2_S, HS^−^, S^2−^ and S_x_^2−^) in solution was measured via the methylene blue method^21,22^. The experiment was performed in triplicate, and the reported results are the average. Details are illustrated in Fig. 1. By proceeding with this experiment, the effect of different pH values on the progress of Eq. (3) would be clear.

**Figure 2:**
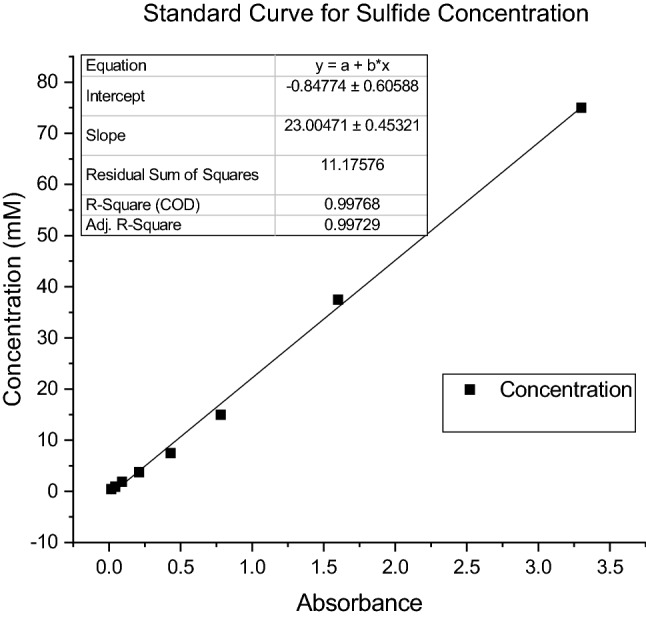
Standard curve for sulfide concentration.

For the methylene blue method, three solutions were prepared. Solution A was a 2% w/v zinc acetate solution in which 2 g of zinc acetate was added to 100 ml of bidistilled water. Solution B, 0.2% dimethyl-*p*-phenylenediamine sulfate solution by which first 20 ml of sulfuric acid 98% was added to 80 ml of bidistilled water and then 0.2 g of dimethyl-*p*-phenylenediamine was added to the solution. Solution C was a 10% FeNH_4_(SO_4_) solution in which 2 ml of 98% sulfuric acid was added to 98 ml of water, and then 10 g of FeNH_4_(SO_4_) was dissolved in the solution. To analyze the sulfide content of a solution, 10 ml of solution A was poured in a flask, and a specific amount of unknown solution was added. Then, 30 ml of water was added, and 5 ml of solution B was poured into the flask. Then, 250 μl of solution B was added, and the flask content reached 50 ml in total by adding the required amount of water. After 20 min, the absorbance of the solution at 670 nm was read by a spectrophotometer (UV mini-1240, Shimadzu Europe, Germany). To calculate the sulfide concentration of unknown solution, the mentioned procedure was developed for different solutions of known concentrations (75, 37.5, 15, 7.5, 3.75 and 1.875 μM), and finally, the standard curve was sketched (Fig. 2).

The other reaction that can occur is the conversion of sulfide ions to thiosulfate without the presence of polysulfide ions and biosulfur (Eq. 4). The existence of these substances intensifies the reaction, which would be analyzed in part two. Herein, the conversion of sulfide ions to thiosulfate was analyzed in the presence of oxygen, and the effect of pH variation and oxygenation on thiosulfate formation was observed.

5 $$2 HS^{ - } + 2 O_{2} \to S_{2} O_{3}^{2 - } + H_{2} O$$

**Figure 3:**
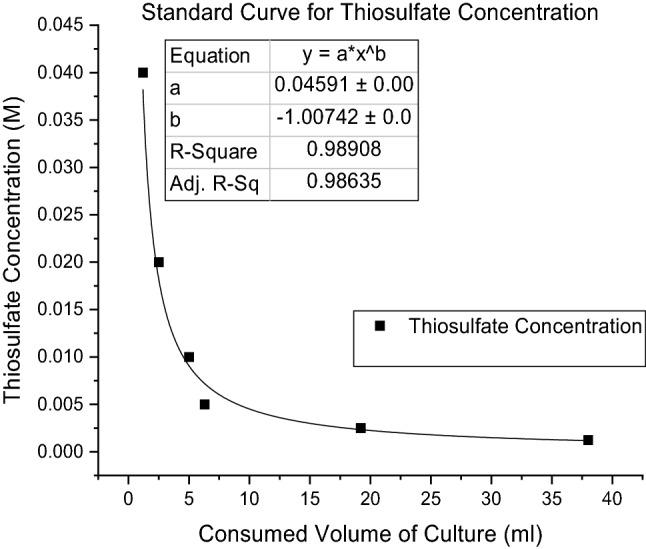
Standard curve for thiosulfate analysis.

To clarify the behavior of this reaction, in the abovementioned six Erlenmeyer flasks, the formation of thiosulfate under two conditions, oxygenated and nonoxygenated, was assessed at six different pH values (pH = 2, 6, 7, 8, 9 and 12) (concentration of formed thiosulfate was measured). Oxygenation was performed by entering a pipeline into the flask, which was connected to high-pressure air. The aeration rate was controlled by a rotameter flow meter (King instrument, USA) to be 5 l/min for 15 min, and dissolved oxygen (DO) was read by a DO meter (Clean DO200, China). To consider the effect of sulfide concentration on thiosulfate formation, the abovementioned experiment was repeated with a high concentration of initial sulfide (0.01 M, 0.5 M, 1 M and 1.5 M). This experiment was performed in triplicate, and the average results were reported. The concentration of thiosulfate ions was analyzed via the iodine titrimetric method^23,24^. In this method, the 0.05 Molar iodine solution was prepared. The titration proceeded with thiosulfate solutions of known concentration, and then the standard curve was sketched for volume (ml) of thiosulfate solution with different dilutions consumed for constant amount of iodine. First, a solution with concentration of 0.04 M thiosulfate (the concentration of thiosulfate in standard medium culture of *Thioalkalivibrio versutus*) was prepared. Then, the solution was diluted by half, and the resulting solution was diluted by half until solutions with concentrations of 0.04, 0.02, 0.01, 0.005, 0.0025 and 0.00125 M thiosulfate were achieved. Then, 1 ml of 0.05 M iodine solution was transferred to an Erlenmeyer flask and diluted with 25 ml of bidistilled water. The resulting iodine solution was titrated with different dilutions of thiosulfate, and for each dilution, the consumed volume was recorded for the brown color of the iodine solution to be colorless. At this point, a few drops of starch solution (0.1 g starch in 100 ml water) were added. Starch is the indicator of iodine by which if there remains any iodine, the solution color turns dark blue. Finally, the standard curve was sketched with the consumed volume of thiosulfate solutions versus solution concentration. The resulting standard curve is illustrated in Fig. 3.

For preparing 0.05 M solution of iodine, 64 g of I_2_ was added to 200 ml of bidistilled water, and then 100 g of KI was added to the solution. The solution is stirred well at 14 °C. The final solution was kept in a dark vessel in a cool place (preferred to be in a refrigerator).

### Part two: thiosulfate formation in de-microbized medium

**Figure 4:**
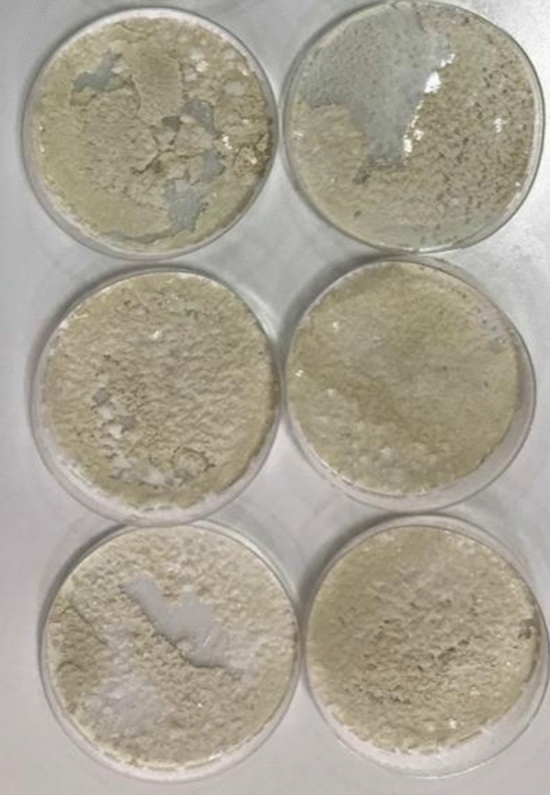
Demicrobized material for abiotic thiosulfate formation experiment.

To better understand the abiotic reactions of sulfide that proceeded in sulfur-oxidizing bacteria medium, a de-microbized medium was prepared, and further experiments were performed to investigate sulfide abiotic behavior. To prepare de-microbized medium, culture medium for *Thioalkalivibrio versutus* (DSMZ, Germany) was prepared, which contained 20 g Na_2_CO_3_, 10 g NaHCO_3_, 5 g NaCl, and 1 g K_2_HPO_4_ in 1000 ml of distilled water that also included 80 mM Na_2_S_2_O_3_, 0.5 mM MgCl_2_, 5 mM KNO_3_ and 2 ml of trace elements. The content of trace elements is described elsewhere^9,25^. *Thioalkalivibrio versutus* (*T. versutus*) was cultivated in 300 ml of culture medium, and growth was monitored by OD_600_ absorbance. As seen in the medium content, the sulfur source is thiosulfate. This experiment aims to determine the effect of sulfide-containing medium on abiotic thiosulfate formation and its effect on process efficiency. As mentioned previously, sulfide ions and biosulfur play roles in the formation of undesired thiosulfate. Therefore, the alluded culture’s thiosulfate content was also monitored every 6 h by the iodine titrimetric method until the thiosulfate finished. At this point, as there is no S source (which is also an energy source) for the bacteria, it is used as an inoculum for the main culture because it contains substances other than thiosulfate. Instead of thiosulfate, one-fourth of the equivalent molar sodium sulfide (0.02 M Na_2_S·3H_2_O) was added. The culture was grown until the sulfide concentration diminished to zero. The total sulfide concentration was monitored by the methylene blue method. When the sulfide was finished, to de-microbize the culture, 400 μg of proteinase K was added to the culture to facilitate cell lysis. The culture medium was poured into petri dishes and placed in an oven at 80 °C for 72 h. After this period, the dishes had lost their water, and the strain was lysed, but biosulfur (biologically produced elemental sulfur) and other medium contents (sulfate, carbonate, bicarbonate, etc.) were remained which is shown in Fig. 4.

The precipitated material in the bottom of the petri dishes was craved gently to an Erlenmeyer flask of 1000 ml that contained 600 ml of bidistilled water. The flask was then placed on a magnetic stirrer/heater at 200 RPM for 72 h. for completely solving the precipitated substances. The resulting solution contains all the components of biological culture other than bacteria. Therefore, there is a solution by which pure abiotic reactions and the behavior of the biological sulfur oxidizing process could be analyzed.

For analyzing the abiotic reactions, it should be mentioned that the main difference between this solution and the Part One experiment is the presence of biosulfur (S^0^). When HS^−^ is added to the solution, in the presence of oxygen, the following reactions can proceed:

6 $$2HS^{ - } + 2S^{0} + 3 O_{2} \to 2S_{2} O_{3}^{2 - } + 2H^{ + }$$

Again, the role of protons is obvious in reaction of Eq. (6), which has a significant effect on the fate of the process. It is worth noting that this reaction is the sum of two reactions in which S_x_^2−^ (polysulfide ion) is formed and consumed:

7-1 $$HS^{ - } + \left( {x - 1} \right) S^{0} \leftrightarrow S_{x}^{2 - } + H^{ + }$$

7-2 $$2 S_{x}^{2 - } + 3 O_{2} \to 2 S_{2} O_{3}^{2 - } + 2 \left( {x - 2} \right) S^{0}$$

To analyze the behavior of the abiotic side of the biodesulfurization process, the de-microbized solution was divided into six Erlenmeyer flasks of equal volume (100 ml in each flask). The pH of the content of each flask was regulated on specific amounts of 2, 6, 7, 8, 9 and 12. The setup of Fig. 1 is attached to each flask, and 1 ml of sulfide stock solution is added to each flask. Released H_2_S was measured in the bottom and top of the flask with a gas analyzer (Gas Clip, USA). Thiosulfate formation was assessed via the iodine titrimetric method. The remaining total sulfide was assessed using the methylene blue method. As in the previous part, the whole experiment was repeated three times, and the results are reported as averages.

### Part three: processing the results by D.X v.13 and *T. versutus* culture under the optimum conditions for the abiotic side

After obtaining the abiotic side of the process and its effect on strain performance, the experiment condition and results of previous sections which were up to 60 experiments, were fed to Design Expert software. The factors and their details are presented in Table 1.

**Table 1:** Details of factors introduced to D. X.

Factor	Name	Type	Subtype	Minimum	Maximum	Coded low	Coded high	Mean	Std. Dev
A	pH	Numeric	Continuous	2	12	− 1 ↔ 2.00	+ 1 ↔ 12.00	7.27	3.05
B	Initial Sulfide	Numeric	Continuous	0.001	1.5	− 1 ↔ 0.00	+ 1 ↔ 2.00	0.6690	0.5801
C	Aeration	Categoric	Nominal	Level 1 of C	Level 2 of C			Levels:	2
D	Biosulfur presence	Categoric	Nominal	Level 1 of D	Level 2 of D			Levels:	2

**Figure 5:**
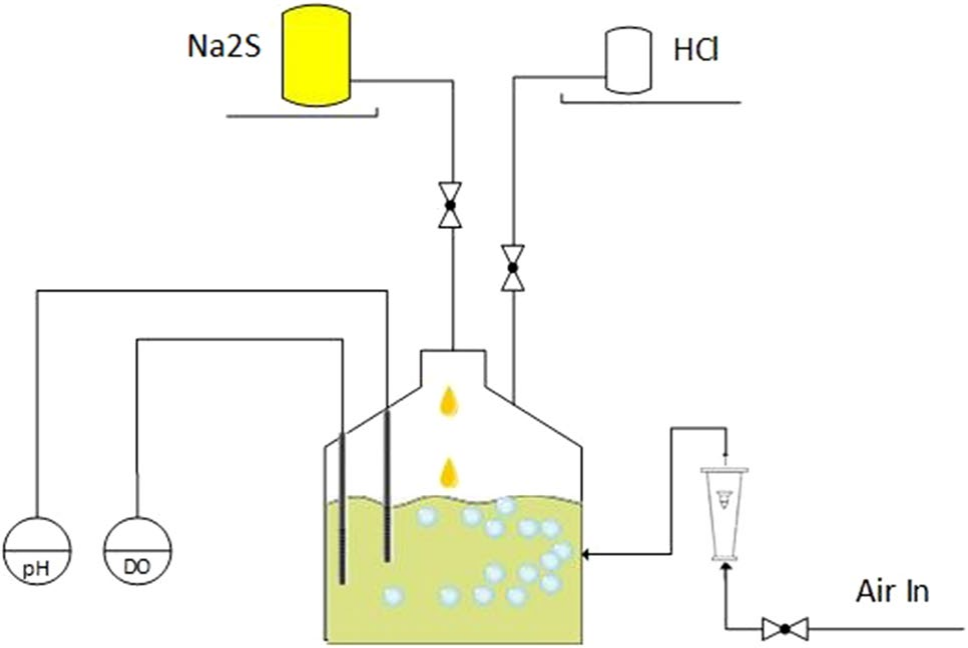
Schematic representation of *T. versutus* culture under the applied conditions for the abiotic side.

As seen in Table 1, two numeric factors, pH and Initial Sulfide and two categoric factor, Aeration and Biosulfur Presence were determined. Aeration and Biosulfur Presence is in two states, level 1 and level 2 by which level 1 indicates that there exists aeration and there exists biosulfur in the medium and level 2 indicates lack of these. Also, the percent of residual sulfide (residual sulfide/initial sulfide) was determined as Response. Then the results of experiments of previous sections (60 experiments) were fed and a statistical model was developed. Using such a model, the optimum abiotic condition with the predicted result for response was obtained. By considering the obtained optimum condition a culture was cultivated by inoculating *T. versutus* in 4 l of the abovementioned medium, which lacks a sulfur source. In the first phase of this experiment, as the capability of sulfide utilization in the culture was low due to the low cell count (which caused the accumulation of sulfide and progression on the abiotic side), sodium sulfide three hydrate at a concentration of 10 g/l was injected into the culture at a rate of 0.5 g/h. After the growth of bacteria, the rate was increased to 1, 1.5 and 2 g/h. In addition to the mentioned culture, a control culture was also cultivated under the same conditions and standard thiosulfate concentrations as the sulfur source. During the culture period, the pH was read continuously and was controlled in the obtained range from previous parts using injection of 5 N HCl. The oxygenation was controlled by using a rotameter flowmeter at 10 l/min, and DO was monitored in case of excess oxygen for better agitation. The vessel (bioreactor) was placed on a magnetic stirrer. In addition to this setup, a similar one was also used as a negative control, by which there was no inoculation. To compare the performance of the controlled abiotic side without controlling conditions, the experiment was repeated, but no control on pH and injection was applied. The setup is given in Fig. 5.

All substances whose origin was not mentioned in the text were purchased from Merckmillipore, Germany.

## Results and discussion

### Effect of proton on H_2_S solubility and release

The solubility of HS^−^ and H_2_S release from the solution was analyzed using the setup illustrated in Fig. 1. In this way, sodium sulfide was introduced to the setup, and the behavior of the solution at various pH values was investigated. The results of H_2_S release versus pH at different times and at both the bottom and top of the vessel are presented in Table 2:

**Table 2:** Released H_2_S at different pH values in different times at top and bottom of the flask.

pH	Detection time (S)	Final time (min)	Final concentration (ppm)		Final pH
			Top	Bottom	
2	43	3	23	54	2.12
6	160	6	4.1	8.6	7.9
7	240	9	0	10	8.6
8	420	20	0	7	9.1
9	–	30	0	0	9.6
12	–	30	0	0	12.2

**Figure 6:**
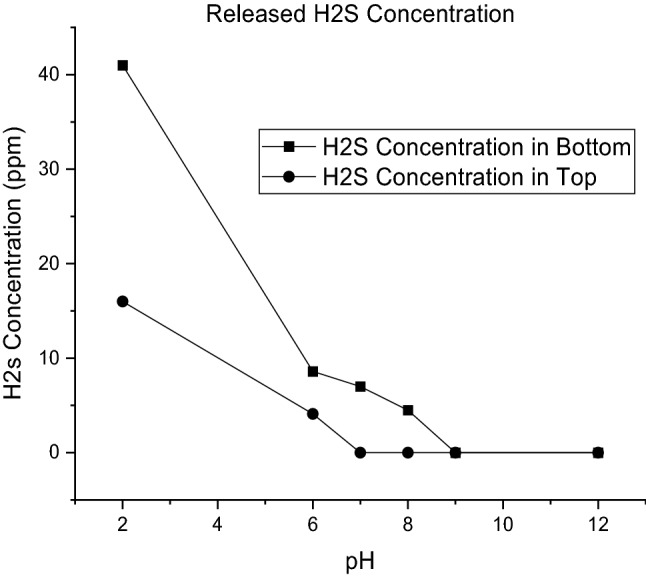
Released H2S concentration after the addition of sodium sulfide at various pH values.

Additionally, the concentrations of released H_2_S at different pH values in the top and bottom of the flasks are shown in Fig. 6.

As shown in Table 2 and Fig. 6, when pH decreases, the amount of released H_2_S increases, and there is a direct relation between proton concentration and H_2_S release. It is because of the equilibrium in Eq. (3). As told before, it is desired to minimize the rate of reaction of Eq. (3), − R_HS rev_. By considering this equation, it should be said that at high concentrations of H^+^, the equilibrium tends to right to consume H^+^ and to diminish Gibbs free energy to zero:

8 $$\Delta G_{2} = \Delta G_{2}^{^\circ } + RT ln K_{2}$$

In this equation, K_2_ is the equilibrium constant of Eq. (3) and could be written as:

9 $$K_{2} = \frac{{\left[ {HS^{ - } } \right]\left[ {H^{ + } } \right]}}{{\left[ {H_{2} S} \right]}}$$

**Figure 7:**
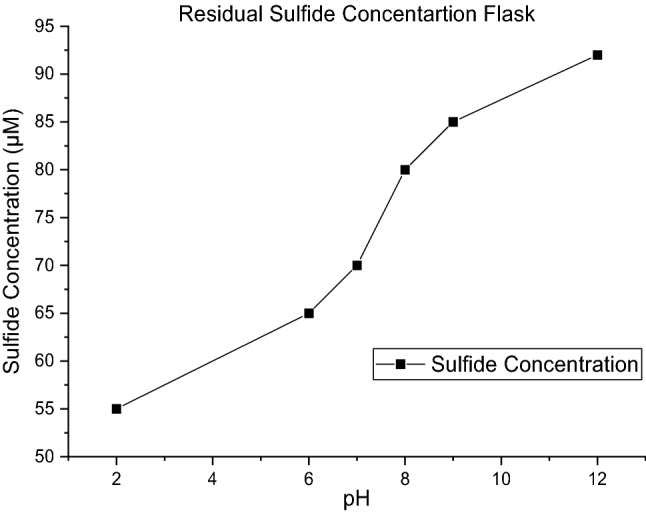
Residual sulfide versus various pH values in sulfide introduced to medium without biosulfur.

In acidic solutions the abiotic reaction (Eq. 3) desires to use proton. In this way, when sodium sulfide is introduced to the solution, because there is no H_2_S and an excess number of protons is present in the environment, the reaction produces hydrogen sulfide until the K_2_ value reaches 1.8. However, in basic solutions where the solution suffers from proton shortage, Eq. (3) does not proceed to the right side because it should save its protons. In the real gas biodesulfurization process, no sodium sulfide or H_2_S is directly introduced to the system. Therefore, in a real process, it is desired to hold the proton content of the solution as low as possible because under such conditions, Eq. (3) tends to the left side, more HS^−^ is produced, which is the desired substance for SOBs. High pH values, in which the solution has low proton content, are desired to avoid the reaction of Eq. (3), the − R_HS rev_ is minimized and saves more sulfide ions for bacteria to produce more elemental sulfur. The results for residual sulfide concentration at various pH values are illustrated in Fig. 7.

As illustrated in Fig. 7, by increasing the pH value, the residual sulfide concentration increases, which means a decrease in the value of − R_HS_ rev. Additionally, it is worth noting that at all pH values in the experiment of part one, no thiosulfate was detected. The reason for such a phenomenon is described in the following section.

### Sulfide oxygenation on different pH values

To investigate the effect of pH, oxygenation, and different sulfide concentrations on thiosulfate formation, the concentration of S_2_O_3_^2−^ was measured in different explained situations. The results are indicated in Table 3.

**Table 3:** Results of the investigation of sulfide oxygenation under different conditions of pH, oxygenation and sulfide concentration.

pH	Sulfide concentration									
	120 µM		0.01 M		0.5 M		1 M		1.5 M	
	O.*	N. O**	O. (µM)	N. O. (µM)	O. (mM)	N. O. (mM)	O. (M)	N. O. (M)	O. (M)	N. O. (M)
2	N. D***	N. D	25	26	22	23	0.04	0.05	0.3	0.4
6	N. D	N. D	26	23	24	21	0.05	0.05	0.3	0.35
7	N. D	N. D	25	28	18	22	0.04	0.05	0.4	0.3
8	N. D	N. D	21	24	22	21	0.06	0.04	0.35	0.23
9	N. D	N. D	26	25	25	19	0.07	0.06	0.43	0.4
12	N. D	N. D	25	27	21	23	0.04	0.05	0.5	0.6

*O.: Stands for oxygenated.

**N. O: Stands for not oxygenated.

***N. D: Stands for Not Detected.

As seen in the results of Table 3 and expected, pH had no effect on the progress of this reaction. In accordance with Eq. (5), it was predictable because there is no role for proton exchange. Therefore, this reaction is independent of pH value. Additionally, oxygenation does not affect the thiosulfate concentration results. This is because the initial DO of the solutions was measured to be approximately 3.6 mg/l. By considering the reaction stoichiometry and inlet sulfide (100 µl of each stock), the oxygen content was in excess in our experiment. However, the concentration of initial sulfide has a significant effect on thiosulfate formation. As seen, by increasing the concentration, the produced thiosulfate increases exponentially, and by decreasing it, thiosulfate decreases at a higher rate. This phenomenon is related to the kinetics of thiosulfate formation from sulfide oxygenation. By considering the following equation for sulfide oxidation, Eq. (10):

10 $$- R_{HS thio1} = R_{thio} = \frac{{d\left[ {Thiosulfate} \right]}}{dt} = k C_{HS}^{\alpha } C_{O2}^{\beta }$$

In this reaction, because the results of our experiments are independent of oxygen concentration, Eq. (10) could be rewritten as:

11 $$R_{thio} = k_{o2} C_{HS}^{\alpha }$$

α is the order of reaction to sulfide ion concentration and is reported to be approximately 1.34^26^. By considering this value, it is expected for concentrations of lower than 1 M to have adverse effects on the reaction rate (Eq. 11) and final concentration of thiosulfate. In lower concentrations, it is intensified. Therefore, for $- R_{HS thio1}$ to be minimal, the concentration of sulfide could be kept as low as possible. To meet this criterion, sulfide should be gradually introduced to the reaction chamber.

### Proton effect on thiosulfate formation in de-microbized medium

**Figure 8:**
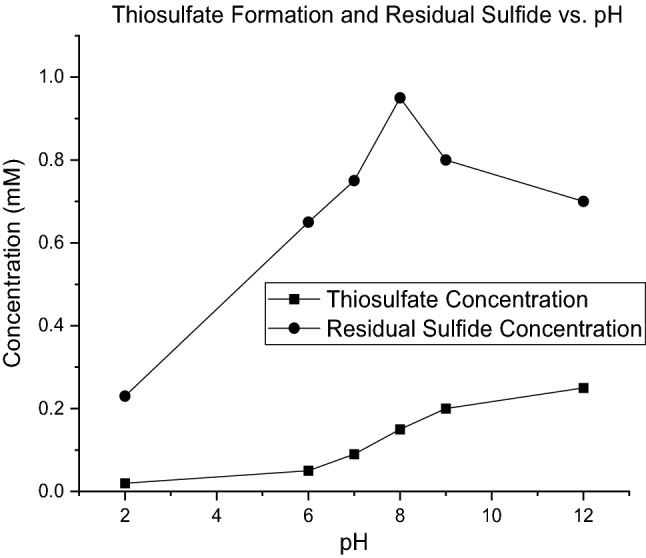
Concentration of produced thiosulfate and residual sulfide in demicrobized medium.

De-microbized medium was prepared as described in detail in the methods section. The aim of preparing such a solution was achieving media with all characteristics of microbial solutions other than living organisms. In other words, pure abiotic media were prepared to analyze the behavioral role of protons. For this reason, sodium sulfide was introduced to such a medium at various initial pH values. As represented in Eq. (6), because of the existence of sulfide, biosulfur and oxygen in the environment, thiosulfate production was expected. Therefore, the concentrations of sulfide and thiosulfate were measured 10 min after injection of sulfide. The results are shown in Fig. 8.

The thiosulfate concentration increases with increasing pH value. These two reactions proceed simultaneously, Eq. (5) that is independent of biosulfur and pH value and dependent just on the concentration of HS^−^ and Eq. (6) by which biosulfur reacts with sulfide and oxygen to produce thiosulfate and release a proton. It is desired to increase the available HS^−^ for the bacterium, and the rate of Eq. (5), − R_HS thio1_ and Rate of Eq. (6), − R_HS thio2_ to be minimized. Equation (5) was analyzed in the previous section. As shown in Fig. 8, to minimize − R_HS thio2,_ it is necessary to keep the pH value as low as possible. As it is seen in Eq. (6), when the proton content of the solution is low, the reaction proceeds to the right to compensate for proton shortage; therefore, more sulfide would be converted to thiosulfate. By considering the results of part one experiment that concluded the pH to be as high as possible and the results of the current experiment that says the pH should be as low as possible, it could be concluded that there is an optimum pH value that is important to be considered to minimize the rate of abiotic sulfide conversion reactions, − R_HS A_. Such pH could result from sketching residual sulfide in de-microbized medium after sulfide injection versus pH values. This is because in de-microbized medium, all three main abiotic reactions of sulfide could occur. As seen in Fig. 8, such a pH could be approximately 8. This pH is not as low as reaction (1) could proceed and not as high as reaction (5) to proceed. Therefore, at this pH, both − R_HS thio1_ and − R_HS thio2_ would be minimized.

### Obtaining optimum abiotic condition and cell culture

To assess the performance of elemental sulfur recovery under the obtained abiotic conditions, a culture for *T. versutus* was cultivated using the setup illustrated in Fig. 5. For obtaining optimum abiotic condition which mean maximum residual sulfide in the culture medium, design expert (D. X v.13) was used. After inserting experiment condition (factors) and results (response) in D. X for 60 experiments, a quadratic model was run. Final equation for model in terms of coded factors are illustrated in Table 4.

**Table 4:** Final equation for model in terms of coded factors.

Residual sulfide	=
0.6583	
0.1352	A
− 0.2297	B
0.0064	C
− 0.1517	D
0.0051	AB
0.0092	AC
0.1388	AD
0.0029	BC
0	BD
0	CD
− 0.0345	A^2^
− 0.057	B^2^

Also, the ANOVA table for the obtained model is illustrated Table 5.

**Table 5:** ANOVA for quadratic model.

Source	Sum of Squares	df	Mean Square	F-value	*p* value	
Model	0.9028	10	0.0903	23.62	< 0.0001	Significant
A-pH	0.0861	1	0.0861	22.52	< 0.0001	
B-Initial Sulfide	0.3507	1	0.3507	91.74	< 0.0001	
C-Aeration	0.0016	1	0.0016	0.428	0.5165	
D-Biosulfur presence	0.2963	1	0.2963	77.52	< 0.0001	

Statistical data of model:

Std. Dev.: 0.0618.

R^2^ = 0.97.

R^2^ Adjusted: 0.92.

Mean = 0.815.

**Figure 9:**
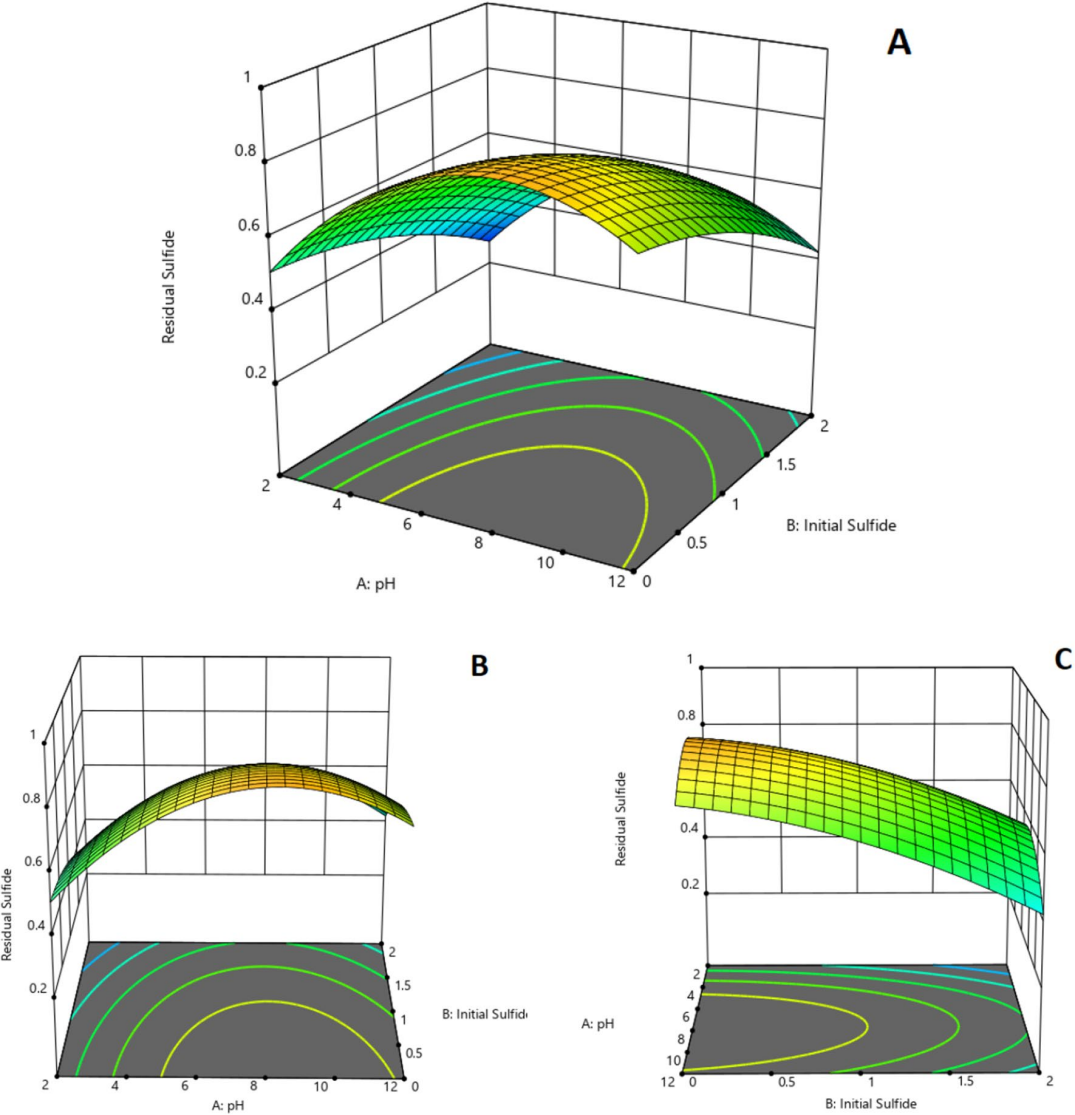
The 3-D plot of resulted model from D. X v.13 which indicates the behavior of response with factors and illustrates optimum condition.

As it is seen from the results of Table 5 with accordance to *p* value and it was also mentioned in results of experiments in previous sections, pH and input sulfide concentration (initial sulfide) has significant effect on the results. This effect was explained in detail in previous sections and the results of model verifies those explanations. The other important factor is Presence of Biosulfur. As this substance is the one of the reactants of Eq. (6), if it could be removed the reaction (6) would not occur any more but as it is obvious, biosulfur of ultimate product of biodesulfurization process and ignoring this component is impossible. This situation could be an initial idea for designing a process in which biosulfur is separated from the culture as it is produced. In such a way, progress of Eq. (6) would be stopped. Designing such a continuous-separation process would be a good idea for future study. The results of model for obtaining optimum abiotic condition and the response to such a condition is extracted from D.X v.13. The 3-D plot of the model from three different views which indicates optimum condition is illustrated in Fig. 9A–C.

**Figure 10:**
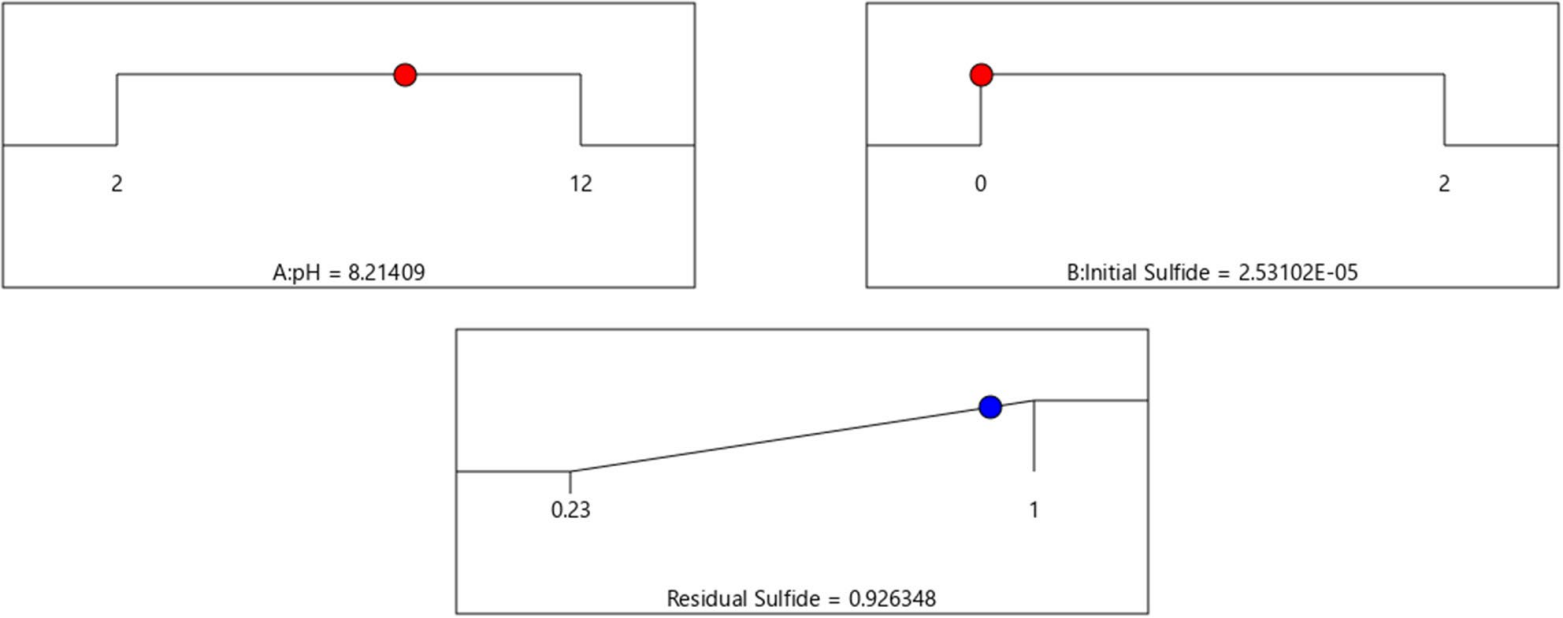
Optimum condition for the obtaining maximum residual sulphide.

As it is illustrated in Fig. 8, the maximum available sulfide for microbe (which is defined as residual sulfide/initial sulfide in the model as response, a value between 0 and 1) is available in low concentrations of sulfide and pH values of around 8. The exact optimum condition and response are available in Fig. 10.

As it is seen in Fig. 10, the optimum residual sulfide would be 92% of input sulfide if pH value be kept in 8.2 and sulfide concentration kept maximally in the range of 2.5E−05 M. For verifying the results of model, the abiotic condition is applied to the culture medium and the results are recorded.

**Figure 11:**
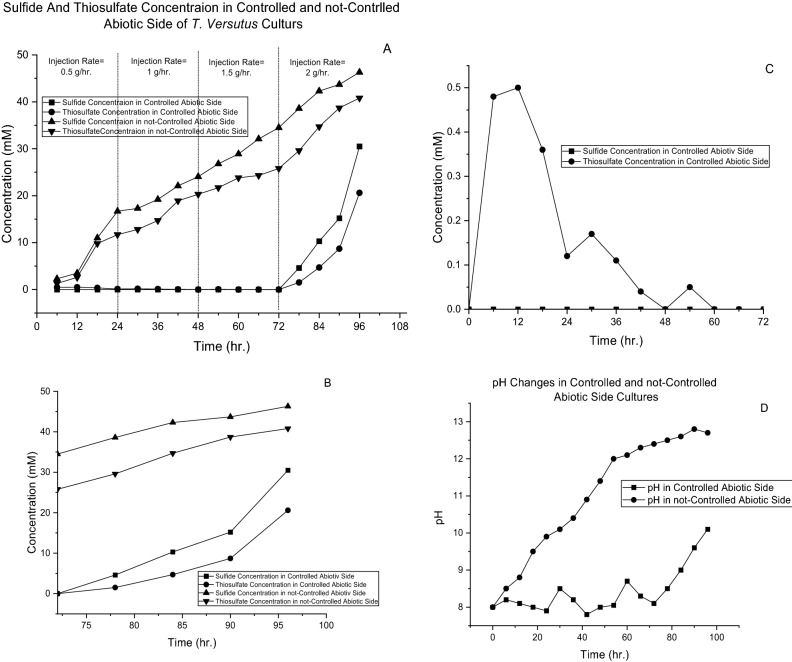
Results of cell culture under obtained abiotic conditions. (**A**) Results of sulfide and thiosulfate concentrations for all 4 days with different injection rates. (**B**) First 3 days of injection up to 1 g/h. (Controlled Abiotic Condition). (**C**) Fourth day of culture with an injection rate of 2 g/h. (Controlled Abiotic Condition. (**D**) pH value in the culture period for the controlled and uncontrolled abiotic sides.

As a result of the model, it is necessary to control the proton content of the solution in which the pH value is kept at 8.2. For this reason, an HCl 5 N solution was prepared and connected to a valve to open if necessary. According to the findings of the previous section, in addition to sulfide toxic effects to cells^27^, to avoid Eq. (5) to progress, it was concluded that sulfide should be introduced to the culture at a slow rate by which the maximum concentration should not exceed 2.5E−05 M. Therefore, a vessel was provided for sodium sulfide with a valve to regulate the injection rate. The initial rate of sulfide injection was set to 0.5 g/h. Because a solution of sodium sulfide produces a hydroxide ion that makes the culture basic and the pH rise, the HCl valve remained open, and the rate was controlled such that the pH remained at approximately 8. As seen in Fig. 5, the pH was read continuously (sample with a controlled abiotic side). On the other setup, there was no control on the abiotic side (pH value and injection rate). During the culture, sulfide and thiosulfate concentrations and pH values were measured every 6 h for both samples, and the rate of sodium sulfide injection increased every 24 h to 1, 1.5, and 2 g/h for former sample (controlled abiotic side sample). The results are represented in Fig. 11.

**Figure 12:**
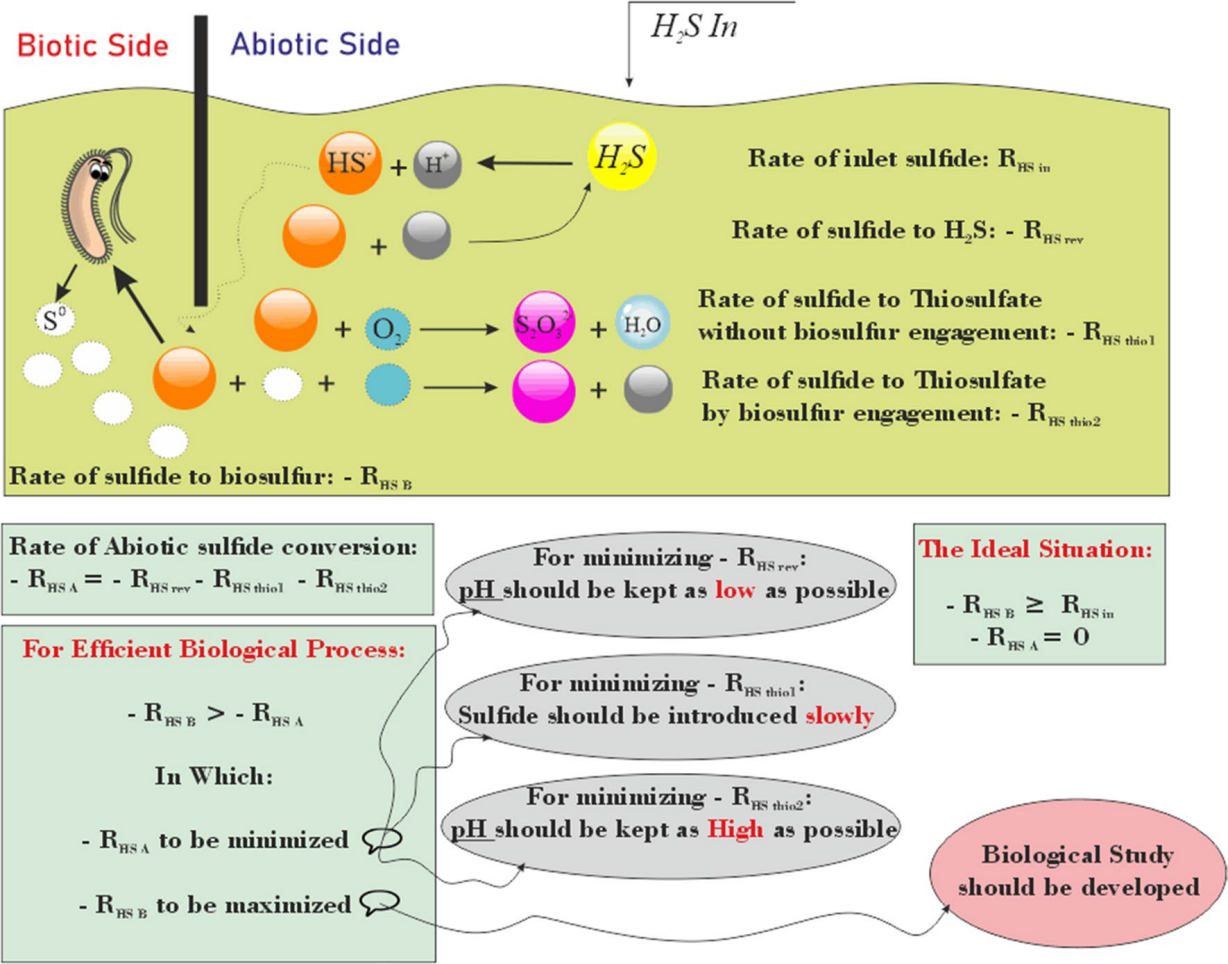
Summery of findings on abiotic side of gas biodesulfurization.

The behavior of the controlled abiotic side culture and noncontrolled abiotic side sample is compared in Fig. 11A and D. As shown in this figure (better observable in Fig. 11B), cultures with a controlled abiotic side had an injection rate of up to 1.5 g/h all sulfide is consumed. In the first 48 h thiosulfate concentration rises to 0.5 mM because of the low count of microbes at the beginning of the culture. Over time, as the cells proliferate, the need for sulfur sources increases, and all sulfides and thiosulfate are utilized by bacteria. After 24 and 48 h, there was a peak because of an increase in the injection rate. As mentioned, the injection rate was increased every 24 h. Therefore, thiosulfate accumulation was slight, which was then utilized by bacteria for proliferation and increasing cell count. At 72 h after cultivation thiosulfate concentration is measured to be 0.12 M, in which by consuming its consumption by cells, the 92% available sulfide for microbe (according to model prediction) was achieved. Everything is ok for injection rates up to 1.5 g/h. As the injection rate increases from 1.5 to 2 g/h. The accumulation of sulfide and thiosulfate was observed (Fig. 11C), and the pH increased (Fig. 11D). Some ulfide is utilized by bacteria, and approximately 40% is converted to thiosulfate. A change in the rate of accumulation of sulfide and thiosulfate was also observed 90 h after culture. This is because of the previous accumulation of sulfide, which has a toxic effect on bacterial performance. Therefore, further accumulation of sulfide and thiosulfate is observed. When the accumulation of sulfide increases, the rate of its conversion to thiosulfate also increases. Additionally, as seen in Fig. 11A from the beginning of the culture, the accumulation of sulfide and thiosulfate occurred in the noncontrolled abiotic side culture. The behavior of this sample is similar to the behavior of the fourth day of sample 1 (controlled abiotic side sample): accumulation of sulfide and thiosulfate and increase of pH value (Fig. 11D). Additionally, no growth was observed in the negative control. Graphical representation of findings on abiotic side of gas biodesulfurization is illustrated in Fig. 12. In this Figure, the optimum abiotic condition explained.

## Conclusion

Biological processes are known to be clean and environmentally friendly processes, but despite this, because of their low efficiency and slow mechanism against chemical processes, applying these processes is limited. One of the useful capabilities of biological processes is utilizing sulfur oxidizing bacteria (SOB) to remove hydrogen sulfide from natural gas. Natural gas biodesulfurization has two sides: one biotic side that contains bacterium performance and one abiotic side that contains chemical reactions that could occur in different situations. For such a biological process to be competitive with its chemical analog process, it is important to upgrade both biological and nonbiological sides. To upgrade sulfur recovery in biological processes, as the main feed of bacteria is HS, it is essential to provide a sufficient amount of sulfide ions to living organisms. Therefore, overcoming the barriers and upgrading the abiotic side precedes the biotic side. Therefore, in the current work, experiments were designed to find the best conditions for the abiotic side of the process. Therefore, it was found that three abiotic reactions consume sulfide to undesired components. One converts it in a reverse reaction to hydrogen sulfide (− R_HS rev_), and two reactions convert it to thiosulfate (− R_HS thio1_ and − R_HS thio2_). For maximum sulfide to be given to the bacteria, it is necessary to minimize these rates. It was found that protons play a vital role in minimizing these rates. Additionally, it was shown that for minimizing − R_HS rev,_ it is essential to keep the pH high. To minimize − R_HS thio1,_ it was concluded that sulfide should be injected into the culture gradually, and the rate of this injection is very important. Additionally, it was shown that for − R_HS thio2_ to be minimal, it is necessary to keep the pH as low as possible. By performing an experiment in de-microbized culture, it was shown that the optimum pH for minimizing − R_HS A_ was 8.2. The cell culture was developed in the setup of Fig. 5, and the rate of sulfide injection was increased up to 1.5 g/h. However, the bacterium was not able to consume an injection rate of 2 g/h. For higher rates of sulfide removal, it is necessary to study the biotic side to increase sulfide removal. It could be done by manipulating the metabolism. Such an action could be developed when there is an accurate understanding of the metabolic pathways which is studied by the group, the report is under edition and would be published later.
